# *fac*-{2-[Bis(2-amino­eth­yl)amino]­ethanaminium}trichloridorhodium(III) chloride hemihydrate

**DOI:** 10.1107/S1600536810051846

**Published:** 2010-12-18

**Authors:** Barbara Kutzky, Christian Neis, Kaspar Hegetschweiler

**Affiliations:** a Fachrichtung Chemie, Universität des Saarlandes, Postfach 151150, D-66041 Saarbrücken, Germany

## Abstract

The crystal structure of the title compound, [Rh(C_6_H_19_N_4_)Cl_3_]Cl·0.5H_2_O, is isotypic with the previously reported Ru analogue. The structure contains two crystallographically independent [Rh(Htren)Cl_3_]^+^ cations with a facial tridentate coordination of the monoprotonated tren ligand [tren = tris­(2-amino­eth­yl)amine], leading to an overall distorted octahedral coordination environment around the Rh(III) atom. In one of the two cations, the ethyl­ene groups of the two chelate rings as well as the non-coordinating ethyl­ammonium group are disordered over two sets of sites [0.579 (3):0.421 (3) occupancy ratio]. A series of N—H⋯Cl and O—H⋯Cl hydrogen bonds stabilizes the structure.

## Related literature

The preparation of the title compound has been described by Hyvärinen *et al.* (2009 ▶) and the crystal structure of the isotypic Ru^III^ complex has been reported by Sakai *et al.* (1996 ▶). Disorder phenomena, caused by a superposition of differently folded chelate rings of the tren ligand have been observed by Düpre *et al.* (1999 ▶). Hypodentate coordination of polyamine ligands has been discussed by Blackman (2005 ▶) and Neis *et al.* (2010 ▶). For disorder phenomena, see: Hirshfeld (1976 ▶).
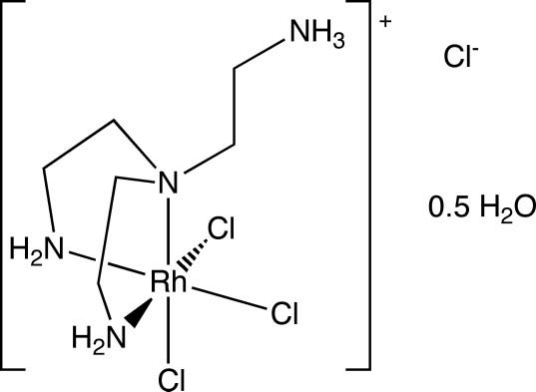


## Experimental

### 

#### Crystal data

[Rh(C_6_H_19_N_4_)Cl_3_]Cl·0.5H_2_O
*M* *_r_* = 400.97
Monoclinic, 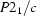

*a* = 13.8022 (6) Å
*b* = 14.1954 (5) Å
*c* = 13.6208 (5) Å
β = 91.196 (1)°
*V* = 2668.11 (18) Å^3^
*Z* = 8
Mo *K*α radiation
μ = 2.06 mm^−1^
*T* = 100 K
0.15 × 0.05 × 0.03 mm

#### Data collection

Bruker X8 APEX KappaCCD diffractometer
Absorption correction: multi-scan (*SADABS*; Bruker, 2010 ▶) *T*_min_ = 0.747, *T*_max_ = 0.941
56384 measured reflections
13775 independent reflections
13026 reflections with *I* > 2σ(*I*)
*R*_int_ = 0.036

#### Refinement

*R*[*F* ^2^ > 2σ(*F* ^2^)] = 0.025
*wR*(*F* ^2^) = 0.065
*S* = 1.18
13775 reflections
328 parameters
2 restraints
H-atom parameters constrained
Δρ_max_ = 1.79 e Å^−3^
Δρ_min_ = −1.89 e Å^−3^

### 

Data collection: *APEX2* (Bruker, 2010 ▶); cell refinement: *SAINT* (Bruker, 2010 ▶); data reduction: *SAINT*; program(s) used to solve structure: *SHELXS97* (Sheldrick, 2008 ▶); program(s) used to refine structure: *SHELXL97* (Sheldrick, 2008 ▶); molecular graphics: *DIAMOND* (Brandenburg, 2010 ▶); software used to prepare material for publication: *SHELXL97* and *PLATON* (Spek, 2009 ▶).

## Supplementary Material

Crystal structure: contains datablocks global, I. DOI: 10.1107/S1600536810051846/si2315sup1.cif

Structure factors: contains datablocks I. DOI: 10.1107/S1600536810051846/si2315Isup2.hkl

Additional supplementary materials:  crystallographic information; 3D view; checkCIF report

## Figures and Tables

**Table 1:** Selected bond lengths (Å)

Rh2—N6	2.0389 (12)
Rh2—N5	2.0510 (12)
Rh2—N7	2.0964 (12)
Rh2—Cl4	2.3522 (3)
Rh2—Cl6	2.3731 (3)
Rh2—Cl5	2.3735 (3)
Rh1—N2	2.0402 (11)
Rh1—N3	2.0420 (11)
Rh1—N1	2.0820 (11)
Rh1—Cl1	2.3626 (3)
Rh1—Cl3	2.3652 (3)
Rh1—Cl2	2.3718 (3)

**Table 2:** Hydrogen-bond geometry (Å, °)

*D*—H⋯*A*	*D*—H	H⋯*A*	*D*⋯*A*	*D*—H⋯*A*
N3—H3*B*⋯Cl7^i^	0.92	2.42	3.2560 (13)	150
N3—H3*A*⋯Cl4^i^	0.92	2.47	3.3279 (12)	155
N2—H2*B*⋯Cl7^i^	0.92	2.34	3.1744 (12)	151
N5—H5*D*⋯Cl8	0.92	2.45	3.3452 (13)	166
N4—H4*A*⋯Cl8^ii^	0.91	2.26	3.1322 (13)	161
N5—H5*C*⋯Cl4^iii^	0.92	2.75	3.5319 (12)	143
N6—H6*D*⋯Cl1^iv^	0.92	2.62	3.4354 (13)	148
N4—H4*C*⋯Cl6^v^	0.91	2.35	3.1729 (13)	150
N81—H81*B*⋯Cl7^vi^	0.91	2.24	3.109 (14)	159
O1*W*—H1*WA*⋯Cl7^vii^	0.86	2.25	3.0797 (15)	163
N2—H2*A*⋯Cl1^viii^	0.92	2.62	3.4348 (12)	148
N6—H6*C*⋯Cl8	0.92	2.36	3.2199 (13)	156

Symmetry codes: (i) 
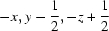
; (ii) 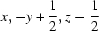; (iii) 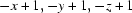; (iv) 
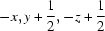
; (v) 
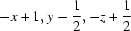
; (vi) 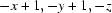; (vii) 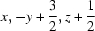; (viii) 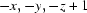.

**Fig. 1.**
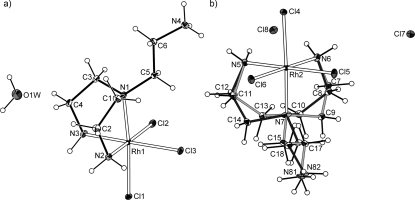
Molecular structure of the title compound. a) The [Rh1Cl3(Htren)]+ cation together with the water molecule; b) the disordered [Rh2Cl3(Htren)]+ cation together with the two chloride counter ions. Displacement ellipsoids are drawn at the 30% probability level.

## supplementary crystallographic information

### Comment

The title compound has recently been obtained as a byproduct in the synthesis of [RhCl_2_(tren)]^+^. Based on the slightly longer wave lengths of the d-d transitions, a partial coordination of the tren ligand was assigned to [RhCl_3_(Htren)]^+^. Considering a step by step binding of the nitrogen donors to a mononuclear aqua-chlorido-Rh^III^ precursor, such an intermediate with three coordinated amino groups could either have a meridional or a facial geometry. The two forms cannot be distinguished by NMR spectroscopy, because both diastereomers exhibit C_s_ symmetry for the averaged solution structure.

The crystal structure analysis confirmed tridentate binding for the Htren^+^ ligand and exhibited a facial geometry for the coordinated diethylenetriamine unit (Fig. 1). Partial metal binding ("hypodentate coordination") of polyamine ligands is well known. Hypodentate coordination can either be caused by the specific steric requirements of the ligand or by slow ligand substitution. The observation, that vigorous conditions in the synthetic procedure resulted in an exclusive formation of [RhCl_2_(tren)]^+^ indicates that the incompletely coordinated ligand of the title compound is due to kinetic rather than thermodynamic reasons. [RhCl_3_(Htren)]^+^ should thus be regarded as an intermediate, trapped on its way to [RhCl_2_(tren)]^+^.

The structure of the title compound is isotypic with the previously reported Ru analogue. The asymmetric unit contains two crystallographically independent [RhCl_3_(Htren)]^+^ cations, two crystallographically independent chloride anions and one water molecule. The entire structure is stabilized by a three dimensional network of hydrogen bonds (Table 2). Notably, one of the hydrogen atoms of the water molecule (H1WB) has no acceptor: its nearest neighbors are the hydrogen atoms of an ethylenediamine group. Two coordinated chloride ions (Cl2 and Cl3) already exhibit O···Cl separations of 3.675 and 3.784 Å. The molecular structure of one of the cations closely approaches C_s_ symmetry with the two chelate rings having a λ and δ conformation. The second cation exhibited some disorder for the five membered Rh—N—C—C—N rings and the non coordinating ethylammonium group. This disorder could be resolved and has been refined as a superposition of two distinct conformers. Within one particular form, the same type of conformation (*i.e.*λ/λ or δ/δ) was observed for the two chelate rings.

In comparison to the isotypic Ru complex, the M—N and M—Cl bonds of the title compound are, as expected, slightly shorter. In both congeners, the M—N bonds of the tertiary nitrogen atoms were slightly longer. In addition, a *trans* influence (push-pull mechanism) has found to be operative (Table 1). However, for the Ru congener, these effects were - if at all - barely significant. This is now different for the title compound, where the accuracy of the structure has been increased by almost one order of magnitude.

### Experimental

Orange crystals of the title compound were grown from aqueous 1 mol/*L* HCl. ^1^H-NMR (D_2_O): δ (p.p.m.) = 3.06 (2*H*), 3.20 (4*H*), 3.35 (2*H*), 3.60 (2*H*), 3.91 (2*H*), 5.52 (broad, 2*NH*), 5.62 (broad, 2*NH*). ^13^C-NMR (D_2_O): δ (p.p.m.)= 35.1, 46.1, 59.6, 62.2. UV/Vis (H_2_O) λ_max_ (nm) = 396, 313.

### Refinement

In the second cation (Rh2), the ethylene groups of both chelate rings as well as the non-coordinating ethylammonium group are disordered, and were considered as a major and minor component with an occupancy of 57.9 (3) % and 42.1 (3) %, respectively. The partially occupied positions of all non hydrogen atoms (major component: C7, C9, C11, C13, C15, C17, N81; minor compounent: C8, C10, C12, C14, C16, C18, N82) could be refined anisotropically. C7 and C8, C11 and C12, N81 and N82 were each refined with equal displacement parameters. However, the disorder obviously generated some inequality of the displacement parameters for neighboring atoms such as N7 and C9 or C14 (Hirshfeld, 1976). H atoms bonded to the water O atom were located in an electron density map and refined with distance restraints of O—H = 0.84 (2) Å, and with *U*_iso_(H) = 1.2*U*_eq_(O). Other H atoms bonded to C- and N-atoms were positioned geometrically and refined using a riding model with free rotation about the C—NH_3_ bond, with C—H = 0.99 Å, N—H = 0.91 (NH_3_ groups) or 0.92 Å (NH_2_ groups), and *U*_iso_(H) = 1.2 or 1.5 (NH_3_ groups) of *U*_eq_ of the pivot atom.

### Crystal data

**Table:**

[Rh(C_6_H_19_N_4_)Cl_3_]Cl·0.5H_2_O	*F*(000) = 1608
*M**_r_* = 400.97	*D*_x_ = 1.996 Mg m^−^^3^
Monoclinic, *P*2_1_/*c*	Mo *K*α radiation, λ = 0.71073 Å
*a* = 13.8022 (6) Å	Cell parameters from 9299 reflections
*b* = 14.1954 (5) Å	θ = 3.2–39.4°
*c* = 13.6208 (5) Å	µ = 2.06 mm^−^^1^
β = 91.196 (1)°	*T* = 100 K
*V* = 2668.11 (18) Å^3^	Prism, orange
*Z* = 8	0.15 × 0.05 × 0.03 mm

### Data collection

**Table:**

Bruker X8 APEX KappaCCD diffractometer	13775 independent reflections
Radiation source: fine-focus sealed tube	13026 reflections with *I* > 2σ(*I*)
graphite	*R*_int_ = 0.036
phi and ω scans	θ_max_ = 37.5°, θ_min_ = 2.1°
Absorption correction: multi-scan (*SADABS*; Bruker, 2010)	*h* = −23→23
*T*_min_ = 0.747, *T*_max_ = 0.941	*k* = −24→24
56384 measured reflections	*l* = −22→18

### Refinement

**Table:**

Refinement on *F*^2^	Primary atom site location: heavy-atom method
Least-squares matrix: full	Secondary atom site location: difference Fourier map
*R*[*F*^2^ > 2σ(*F*^2^)] = 0.025	Hydrogen site location: inferred from neighbouring sites
*wR*(*F*^2^) = 0.065	H-atom parameters constrained
*S* = 1.18	*w* = 1/[σ^2^(*F*_o_^2^) + (0.0142*P*)^2^ + 3.4613*P*] where *P* = (*F*_o_^2^ + 2*F*_c_^2^)/3
13775 reflections	(Δ/σ)_max_ = 0.001
328 parameters	Δρ_max_ = 1.79 e Å^−^^3^
2 restraints	Δρ_min_ = −1.89 e Å^−^^3^

### Special details

**Table:**

Geometry. All e.s.d.'s (except the e.s.d. in the dihedral angle between two l.s. planes) are estimated using the full covariance matrix. The cell e.s.d.'s are taken into account individually in the estimation of e.s.d.'s in distances, angles and torsion angles; correlations between e.s.d.'s in cell parameters are only used when they are defined by crystal symmetry. An approximate (isotropic) treatment of cell e.s.d.'s is used for estimating e.s.d.'s involving l.s. planes.
Refinement. Refinement of *F*^2^ against ALL reflections. The weighted *R*-factor *wR* and goodness of fit *S* are based on *F*^2^, conventional *R*-factors *R* are based on *F*, with *F* set to zero for negative *F*^2^. The threshold expression of *F*^2^ > σ(*F*^2^) is used only for calculating *R*-factors(gt) *etc*. and is not relevant to the choice of reflections for refinement. *R*-factors based on *F*^2^ are statistically about twice as large as those based on *F*, and *R*- factors based on ALL data will be even larger.

### Fractional atomic coordinates and isotropic or equivalent isotropic displacement parameters (Å^2^)

**Table:**

	*x*	*y*	*z*	*U*_iso_*/*U*_eq_	Occ. (<1)
Rh2	0.456131 (7)	0.457663 (7)	0.295722 (7)	0.00764 (2)	
Cl4	0.35083 (2)	0.54510 (2)	0.39418 (2)	0.01355 (5)	
Cl5	0.41181 (3)	0.55130 (2)	0.15758 (3)	0.01525 (6)	
Cl6	0.58495 (2)	0.55887 (2)	0.34534 (3)	0.01457 (5)	
N5	0.49273 (8)	0.37552 (8)	0.41469 (9)	0.01239 (18)	
H5C	0.5065	0.4125	0.4687	0.015*	
H5D	0.4426	0.3355	0.4295	0.015*	
N6	0.34500 (9)	0.37247 (9)	0.25038 (9)	0.01339 (18)	
H6C	0.3199	0.3433	0.3045	0.016*	
H6D	0.2968	0.4094	0.2230	0.016*	
N7	0.54171 (10)	0.36156 (9)	0.21917 (10)	0.0158 (2)	
N81	0.7151 (7)	0.4973 (11)	0.0537 (10)	0.0137 (10)	0.579 (3)
H81A	0.7110	0.5503	0.0162	0.021*	0.579 (3)
H81B	0.7268	0.4467	0.0146	0.021*	0.579 (3)
H81C	0.7643	0.5036	0.0988	0.021*	0.579 (3)
C11	0.5795 (8)	0.3211 (6)	0.3865 (7)	0.0144 (8)	0.579 (3)
H11A	0.5909	0.2688	0.4335	0.017*	0.579 (3)
H11B	0.6373	0.3624	0.3878	0.017*	0.579 (3)
C7	0.3707 (4)	0.3016 (5)	0.1806 (5)	0.0133 (6)	0.579 (3)
H7A	0.3202	0.2980	0.1280	0.016*	0.579 (3)
H7B	0.3744	0.2395	0.2135	0.016*	0.579 (3)
C9	0.46844 (17)	0.32497 (16)	0.13587 (17)	0.0126 (4)	0.579 (3)
H9A	0.4597	0.3741	0.0848	0.015*	0.579 (3)
H9B	0.4953	0.2681	0.1044	0.015*	0.579 (3)
C13	0.56229 (18)	0.28239 (16)	0.28408 (18)	0.0134 (4)	0.579 (3)
H13A	0.6205	0.2482	0.2621	0.016*	0.579 (3)
H13B	0.5069	0.2381	0.2835	0.016*	0.579 (3)
C15	0.62883 (18)	0.39463 (17)	0.1702 (2)	0.0119 (4)	0.579 (3)
H15A	0.6525	0.3428	0.1285	0.014*	0.579 (3)
H15B	0.6793	0.4066	0.2213	0.014*	0.579 (3)
C17	0.61978 (18)	0.48254 (17)	0.1066 (2)	0.0123 (4)	0.579 (3)
H17A	0.6057	0.5379	0.1481	0.015*	0.579 (3)
H17B	0.5660	0.4749	0.0581	0.015*	0.579 (3)
N82	0.7010 (11)	0.5007 (16)	0.0506 (15)	0.0137 (10)	0.421 (3)
H82A	0.7544	0.4635	0.0456	0.021*	0.421 (3)
H82B	0.7189	0.5623	0.0482	0.021*	0.421 (3)
H82C	0.6589	0.4879	0.0001	0.021*	0.421 (3)
C12	0.5738 (11)	0.3072 (9)	0.3985 (10)	0.0144 (8)	0.421 (3)
H12A	0.5479	0.2422	0.3950	0.017*	0.421 (3)
H12B	0.6211	0.3105	0.4541	0.017*	0.421 (3)
C8	0.3877 (6)	0.3096 (7)	0.1675 (7)	0.0133 (6)	0.421 (3)
H8A	0.3451	0.2545	0.1552	0.016*	0.421 (3)
H8B	0.3917	0.3462	0.1058	0.016*	0.421 (3)
C10	0.4876 (2)	0.2769 (2)	0.2001 (3)	0.0139 (6)	0.421 (3)
H10A	0.4841	0.2379	0.2601	0.017*	0.421 (3)
H10B	0.5182	0.2394	0.1478	0.017*	0.421 (3)
C14	0.6233 (2)	0.3313 (2)	0.3038 (2)	0.0136 (5)	0.421 (3)
H14A	0.6606	0.2762	0.2810	0.016*	0.421 (3)
H14B	0.6692	0.3840	0.3152	0.016*	0.421 (3)
C16	0.5993 (3)	0.3907 (2)	0.1350 (3)	0.0132 (5)	0.421 (3)
H16A	0.5551	0.3949	0.0770	0.020*	0.421 (3)
H16B	0.6463	0.3398	0.1217	0.020*	0.421 (3)
C18	0.6550 (3)	0.4822 (2)	0.1422 (3)	0.0135 (6)	0.421 (3)
H18A	0.6103	0.5343	0.1578	0.020*	0.421 (3)
H18B	0.7045	0.4779	0.1957	0.020*	0.421 (3)
Rh1	−0.050193 (6)	0.022538 (6)	0.294250 (7)	0.00769 (2)	
Cl1	−0.14238 (2)	−0.08013 (2)	0.39188 (2)	0.01218 (5)	
Cl2	−0.08326 (2)	−0.07112 (2)	0.15330 (2)	0.01352 (5)	
Cl3	0.09065 (2)	−0.06574 (2)	0.33366 (3)	0.01344 (5)	
N1	0.02397 (8)	0.12427 (8)	0.21533 (9)	0.01062 (16)	
N2	−0.01835 (8)	0.10429 (8)	0.41382 (9)	0.01167 (17)	
H2B	−0.0739	0.1151	0.4482	0.014*	
H2A	0.0249	0.0730	0.4545	0.014*	
N3	−0.17010 (8)	0.10130 (8)	0.26043 (9)	0.01160 (17)	
H3A	−0.2102	0.0679	0.2184	0.014*	
H3B	−0.2036	0.1133	0.3168	0.014*	
N4	0.22326 (9)	0.09829 (9)	0.02885 (9)	0.01396 (19)	
H4A	0.2549	0.1396	−0.0103	0.017*	
H4B	0.1881	0.0575	−0.0091	0.017*	
H4C	0.2672	0.0656	0.0662	0.017*	
C5	0.09455 (9)	0.08094 (9)	0.14742 (10)	0.01189 (19)	
H5A	0.0583	0.0427	0.0983	0.014*	
H5B	0.1372	0.0377	0.1856	0.014*	
C1	0.07923 (11)	0.18286 (10)	0.28988 (11)	0.0156 (2)	
H1A	0.0927	0.2455	0.2614	0.019*	
H1B	0.1421	0.1522	0.3052	0.019*	
C3	−0.05095 (10)	0.17878 (10)	0.15695 (11)	0.0154 (2)	
H3C	−0.0661	0.1448	0.0950	0.018*	
H3D	−0.0243	0.2413	0.1398	0.018*	
C4	−0.14309 (10)	0.19188 (10)	0.21365 (12)	0.0155 (2)	
H4D	−0.1331	0.2408	0.2647	0.019*	
H4E	−0.1960	0.2129	0.1686	0.019*	
C2	0.02394 (11)	0.19537 (10)	0.38390 (11)	0.0162 (2)	
H2C	0.0682	0.2189	0.4365	0.019*	
H2D	−0.0283	0.2423	0.3736	0.019*	
C6	0.15742 (12)	0.15079 (10)	0.09370 (12)	0.0185 (3)	
H6A	0.1162	0.1941	0.0541	0.022*	
H6B	0.1957	0.1887	0.1416	0.022*	
O1W	0.16704 (14)	0.59698 (11)	0.57048 (15)	0.0440 (5)	
H1WA	0.1880	0.6496	0.5480	0.053*	
H1WB	0.1240	0.6050	0.6150	0.053*	
Cl7	0.21905 (2)	0.69611 (2)	0.02866 (3)	0.01468 (5)	
Cl8	0.30749 (3)	0.22405 (2)	0.42426 (3)	0.01559 (6)	

### Atomic displacement parameters (Å^2^)

**Table:**

	*U*^11^	*U*^22^	*U*^33^	*U*^12^	*U*^13^	*U*^23^
Rh2	0.00869 (4)	0.00589 (4)	0.00829 (4)	−0.00005 (2)	−0.00082 (3)	0.00014 (2)
Cl4	0.01259 (11)	0.01427 (12)	0.01383 (13)	0.00339 (9)	0.00147 (9)	−0.00073 (9)
Cl5	0.01943 (14)	0.01270 (12)	0.01342 (13)	−0.00283 (10)	−0.00451 (10)	0.00451 (9)
Cl6	0.01406 (12)	0.01173 (12)	0.01772 (14)	−0.00389 (9)	−0.00425 (10)	0.00080 (10)
N5	0.0131 (4)	0.0113 (4)	0.0128 (5)	0.0018 (3)	−0.0008 (3)	0.0020 (3)
N6	0.0156 (5)	0.0122 (4)	0.0123 (5)	−0.0047 (4)	−0.0014 (3)	0.0008 (3)
N7	0.0212 (5)	0.0079 (4)	0.0188 (5)	−0.0005 (4)	0.0093 (4)	−0.0007 (4)
N81	0.008 (3)	0.0135 (11)	0.0201 (10)	0.002 (2)	0.0063 (18)	0.0006 (8)
C11	0.0156 (13)	0.010 (2)	0.017 (2)	0.0036 (15)	−0.0036 (14)	0.0029 (14)
C7	0.015 (2)	0.0123 (13)	0.0129 (18)	−0.0029 (13)	0.0009 (12)	−0.0023 (11)
C9	0.0148 (9)	0.0105 (8)	0.0126 (9)	−0.0014 (6)	0.0004 (7)	−0.0029 (6)
C13	0.0163 (9)	0.0082 (8)	0.0157 (9)	0.0034 (7)	0.0010 (7)	0.0024 (7)
C15	0.0104 (9)	0.0115 (9)	0.0140 (10)	0.0025 (7)	0.0020 (8)	0.0018 (7)
C17	0.0097 (8)	0.0128 (9)	0.0144 (10)	0.0011 (6)	0.0019 (8)	0.0032 (7)
N82	0.008 (3)	0.0135 (11)	0.0201 (10)	0.002 (2)	0.0063 (18)	0.0006 (8)
C12	0.0156 (13)	0.010 (2)	0.017 (2)	0.0036 (15)	−0.0036 (14)	0.0029 (14)
C8	0.015 (2)	0.0123 (13)	0.0129 (18)	−0.0029 (13)	0.0009 (12)	−0.0023 (11)
C10	0.0157 (12)	0.0090 (11)	0.0170 (13)	−0.0014 (9)	−0.0004 (10)	−0.0027 (9)
C14	0.0126 (11)	0.0119 (12)	0.0161 (13)	0.0042 (9)	−0.0012 (9)	0.0003 (9)
C16	0.0155 (14)	0.0123 (12)	0.0118 (14)	0.0002 (10)	0.0027 (11)	−0.0015 (10)
C18	0.0141 (13)	0.0136 (13)	0.0129 (13)	−0.0024 (10)	0.0019 (11)	−0.0018 (10)
Rh1	0.00765 (4)	0.00523 (4)	0.01013 (4)	−0.00032 (2)	−0.00141 (3)	−0.00011 (2)
Cl1	0.01169 (11)	0.01046 (11)	0.01437 (12)	−0.00173 (9)	0.00012 (9)	0.00220 (9)
Cl2	0.01728 (13)	0.00891 (11)	0.01422 (13)	−0.00154 (9)	−0.00346 (10)	−0.00250 (9)
Cl3	0.01081 (11)	0.01042 (11)	0.01899 (14)	0.00193 (9)	−0.00234 (9)	0.00180 (10)
N1	0.0113 (4)	0.0078 (4)	0.0128 (4)	−0.0003 (3)	0.0010 (3)	0.0000 (3)
N2	0.0125 (4)	0.0100 (4)	0.0125 (4)	−0.0010 (3)	−0.0008 (3)	−0.0010 (3)
N3	0.0106 (4)	0.0095 (4)	0.0146 (5)	0.0004 (3)	−0.0017 (3)	0.0008 (3)
N4	0.0116 (4)	0.0160 (5)	0.0143 (5)	0.0001 (4)	0.0015 (3)	−0.0005 (4)
C5	0.0125 (5)	0.0092 (4)	0.0141 (5)	−0.0001 (4)	0.0016 (4)	0.0001 (4)
C1	0.0175 (5)	0.0130 (5)	0.0163 (6)	−0.0067 (4)	0.0022 (4)	−0.0040 (4)
C3	0.0162 (5)	0.0126 (5)	0.0174 (6)	0.0032 (4)	0.0019 (4)	0.0050 (4)
C4	0.0149 (5)	0.0109 (5)	0.0208 (6)	0.0039 (4)	0.0019 (4)	0.0040 (4)
C2	0.0211 (6)	0.0117 (5)	0.0159 (6)	−0.0053 (4)	0.0022 (4)	−0.0041 (4)
C6	0.0210 (6)	0.0113 (5)	0.0235 (7)	0.0002 (4)	0.0103 (5)	0.0014 (5)
O1W	0.0534 (10)	0.0139 (6)	0.0662 (12)	−0.0023 (6)	0.0344 (9)	0.0031 (7)
Cl7	0.01501 (12)	0.01145 (12)	0.01767 (14)	−0.00112 (9)	0.00213 (10)	−0.00116 (10)
Cl8	0.02018 (14)	0.01051 (12)	0.01618 (13)	−0.00098 (10)	0.00267 (10)	0.00115 (10)

### Geometric parameters (Å, °)

**Table:**

Rh2—N6	2.0389 (12)	C8—H8A	0.9900
Rh2—N5	2.0510 (12)	C8—H8B	0.9900
Rh2—N7	2.0964 (12)	C10—H10A	0.9900
Rh2—Cl4	2.3522 (3)	C10—H10B	0.9900
Rh2—Cl6	2.3731 (3)	C14—H14A	0.9900
Rh2—Cl5	2.3735 (3)	C14—H14B	0.9900
N5—C11	1.482 (12)	C16—C18	1.511 (5)
N5—C12	1.501 (17)	C16—H16A	0.9900
N5—H5C	0.9200	C16—H16B	0.9900
N5—H5D	0.9200	C18—H18A	0.9900
N6—C7	1.434 (7)	C18—H18B	0.9900
N6—C8	1.564 (10)	Rh1—N2	2.0402 (11)
N6—H6C	0.9200	Rh1—N3	2.0420 (11)
N6—H6D	0.9200	Rh1—N1	2.0820 (11)
N7—C10	1.435 (3)	Rh1—Cl1	2.3626 (3)
N7—C13	1.454 (3)	Rh1—Cl3	2.3652 (3)
N7—C15	1.465 (3)	Rh1—Cl2	2.3718 (3)
N7—C16	1.468 (4)	N1—C5	1.4901 (18)
N7—C9	1.591 (3)	N1—C3	1.5054 (18)
N7—C14	1.652 (3)	N1—C1	1.5074 (18)
N81—C17	1.527 (13)	N2—C2	1.4793 (18)
N81—H81A	0.9100	N2—H2B	0.9200
N81—H81B	0.9100	N2—H2A	0.9200
N81—H81C	0.9100	N3—C4	1.4860 (18)
C11—C13	1.514 (11)	N3—H3A	0.9200
C11—H11A	0.9900	N3—H3B	0.9200
C11—H11B	0.9900	N4—C6	1.4816 (19)
C7—C9	1.529 (5)	N4—H4A	0.9100
C7—H7A	0.9900	N4—H4B	0.9100
C7—H7B	0.9900	N4—H4C	0.9100
C9—H9A	0.9900	C5—C6	1.516 (2)
C9—H9B	0.9900	C5—H5A	0.9900
C13—H13A	0.9900	C5—H5B	0.9900
C13—H13B	0.9900	C1—C2	1.515 (2)
C15—C17	1.522 (3)	C1—H1A	0.9900
C15—H15A	0.9900	C1—H1B	0.9900
C15—H15B	0.9900	C3—C4	1.513 (2)
C17—H17A	0.9900	C3—H3C	0.9900
C17—H17B	0.9900	C3—H3D	0.9900
N82—C18	1.44 (2)	C4—H4D	0.9900
N82—H82A	0.9100	C4—H4E	0.9900
N82—H82B	0.9100	C2—H2C	0.9900
N82—H82C	0.9100	C2—H2D	0.9900
C12—C14	1.511 (13)	C6—H6A	0.9900
C12—H12A	0.9900	C6—H6B	0.9900
C12—H12B	0.9900	O1W—H1WA	0.8603
C8—C10	1.513 (11)	O1W—H1WB	0.8650
			
N6—Rh2—N5	94.21 (5)	N6—C8—H8B	110.0
N6—Rh2—N7	83.77 (5)	H8A—C8—H8B	108.4
N5—Rh2—N7	83.74 (5)	N7—C10—C8	105.3 (4)
N6—Rh2—Cl4	90.90 (4)	N7—C10—H10A	110.7
N5—Rh2—Cl4	89.68 (3)	C8—C10—H10A	110.7
N7—Rh2—Cl4	171.19 (3)	N7—C10—H10B	110.7
N6—Rh2—Cl6	178.74 (4)	C8—C10—H10B	110.7
N5—Rh2—Cl6	87.05 (3)	H10A—C10—H10B	108.8
N7—Rh2—Cl6	96.26 (4)	C12—C14—N7	109.9 (6)
Cl4—Rh2—Cl6	89.216 (13)	C12—C14—H14A	109.7
N6—Rh2—Cl5	84.96 (4)	N7—C14—H14A	109.7
N5—Rh2—Cl5	179.17 (4)	C12—C14—H14B	109.7
N7—Rh2—Cl5	96.24 (4)	N7—C14—H14B	109.7
Cl4—Rh2—Cl5	90.250 (13)	H14A—C14—H14B	108.2
Cl6—Rh2—Cl5	93.777 (12)	N7—C16—C18	118.3 (3)
C11—N5—Rh2	106.2 (3)	N7—C16—H16A	107.7
C12—N5—Rh2	115.1 (5)	C18—C16—H16A	107.7
C11—N5—H5C	110.5	N7—C16—H16B	107.7
C12—N5—H5C	110.1	C18—C16—H16B	107.7
Rh2—N5—H5C	110.5	H16A—C16—H16B	107.1
C11—N5—H5D	110.5	N82—C18—C16	109.5 (9)
C12—N5—H5D	101.5	N82—C18—H18A	109.8
Rh2—N5—H5D	110.5	C16—C18—H18A	109.8
H5C—N5—H5D	108.7	N82—C18—H18B	109.8
C7—N6—Rh2	115.0 (2)	C16—C18—H18B	109.8
C8—N6—Rh2	105.4 (3)	H18A—C18—H18B	108.2
C7—N6—H6C	108.5	N2—Rh1—N3	91.65 (5)
C8—N6—H6C	118.4	N2—Rh1—N1	85.27 (5)
Rh2—N6—H6C	108.5	N3—Rh1—N1	84.76 (5)
C7—N6—H6D	108.5	N2—Rh1—Cl1	90.64 (3)
C8—N6—H6D	108.2	N3—Rh1—Cl1	91.22 (3)
Rh2—N6—H6D	108.5	N1—Rh1—Cl1	174.17 (3)
H6C—N6—H6D	107.5	N2—Rh1—Cl3	87.58 (3)
C13—N7—C15	111.90 (16)	N3—Rh1—Cl3	178.79 (3)
C10—N7—C16	112.5 (2)	N1—Rh1—Cl3	94.25 (3)
C13—N7—C9	107.12 (15)	Cl1—Rh1—Cl3	89.722 (12)
C15—N7—C9	107.18 (17)	N2—Rh1—Cl2	178.45 (3)
C10—N7—C14	104.7 (2)	N3—Rh1—Cl2	89.02 (3)
C16—N7—C14	104.2 (2)	N1—Rh1—Cl2	93.40 (3)
C10—N7—Rh2	109.80 (15)	Cl1—Rh1—Cl2	90.746 (12)
C13—N7—Rh2	107.78 (12)	Cl3—Rh1—Cl2	91.723 (12)
C15—N7—Rh2	119.63 (12)	C5—N1—C3	109.57 (11)
C16—N7—Rh2	121.67 (15)	C5—N1—C1	108.53 (10)
C9—N7—Rh2	102.17 (11)	C3—N1—C1	113.87 (11)
C14—N7—Rh2	101.84 (13)	C5—N1—Rh1	111.65 (8)
C17—N81—H81A	109.5	C3—N1—Rh1	106.83 (8)
C17—N81—H81B	109.5	C1—N1—Rh1	106.39 (8)
H81A—N81—H81B	109.5	C2—N2—Rh1	110.91 (9)
C17—N81—H81C	109.5	C2—N2—H2B	109.5
H81A—N81—H81C	109.5	Rh1—N2—H2B	109.5
H81B—N81—H81C	109.5	C2—N2—H2A	109.5
N5—C11—C13	108.4 (6)	Rh1—N2—H2A	109.5
N5—C11—H11A	110.0	H2B—N2—H2A	108.0
C13—C11—H11A	110.0	C4—N3—Rh1	111.17 (8)
N5—C11—H11B	110.0	C4—N3—H3A	109.4
C13—C11—H11B	110.0	Rh1—N3—H3A	109.4
H11A—C11—H11B	108.4	C4—N3—H3B	109.4
N6—C7—C9	110.1 (4)	Rh1—N3—H3B	109.4
N6—C7—H7A	109.6	H3A—N3—H3B	108.0
C9—C7—H7A	109.6	C6—N4—H4A	109.5
N6—C7—H7B	109.6	C6—N4—H4B	109.5
C9—C7—H7B	109.6	H4A—N4—H4B	109.5
H7A—C7—H7B	108.1	C6—N4—H4C	109.5
C7—C9—N7	109.9 (3)	H4A—N4—H4C	109.5
C7—C9—H9A	109.7	H4B—N4—H4C	109.5
N7—C9—H9A	109.7	N1—C5—C6	114.70 (11)
C7—C9—H9B	109.7	N1—C5—H5A	108.6
N7—C9—H9B	109.7	C6—C5—H5A	108.6
H9A—C9—H9B	108.2	N1—C5—H5B	108.6
N7—C13—C11	107.7 (3)	C6—C5—H5B	108.6
N7—C13—H13A	110.2	H5A—C5—H5B	107.6
C11—C13—H13A	110.2	N1—C1—C2	112.17 (11)
N7—C13—H13B	110.2	N1—C1—H1A	109.2
C11—C13—H13B	110.2	C2—C1—H1A	109.2
H13A—C13—H13B	108.5	N1—C1—H1B	109.2
N7—C15—C17	117.61 (19)	C2—C1—H1B	109.2
N7—C15—H15A	107.9	H1A—C1—H1B	107.9
C17—C15—H15A	107.9	N1—C3—C4	111.65 (11)
N7—C15—H15B	107.9	N1—C3—H3C	109.3
C17—C15—H15B	107.9	C4—C3—H3C	109.3
H15A—C15—H15B	107.2	N1—C3—H3D	109.3
C15—C17—N81	108.6 (6)	C4—C3—H3D	109.3
C15—C17—H17A	110.0	H3C—C3—H3D	108.0
N81—C17—H17A	110.0	N3—C4—C3	109.49 (11)
C15—C17—H17B	110.0	N3—C4—H4D	109.8
N81—C17—H17B	110.0	C3—C4—H4D	109.8
H17A—C17—H17B	108.3	N3—C4—H4E	109.8
C18—N82—H82A	109.5	C3—C4—H4E	109.8
C18—N82—H82B	109.5	H4D—C4—H4E	108.2
H82A—N82—H82B	109.5	N2—C2—C1	109.82 (11)
C18—N82—H82C	109.5	N2—C2—H2C	109.7
H82A—N82—H82C	109.5	C1—C2—H2C	109.7
H82B—N82—H82C	109.5	N2—C2—H2D	109.7
N5—C12—C14	109.3 (7)	C1—C2—H2D	109.7
N5—C12—H12A	109.8	H2C—C2—H2D	108.2
C14—C12—H12A	109.8	N4—C6—C5	108.85 (12)
N5—C12—H12B	109.8	N4—C6—H6A	109.9
C14—C12—H12B	109.8	C5—C6—H6A	109.9
H12A—C12—H12B	108.3	N4—C6—H6B	109.9
C10—C8—N6	108.5 (7)	C5—C6—H6B	109.9
C10—C8—H8A	110.0	H6A—C6—H6B	108.3
N6—C8—H8A	110.0	H1WA—O1W—H1WB	112.1
C10—C8—H8B	110.0		
			
N6—Rh2—N5—C11	102.0 (4)	Rh2—N7—C15—C17	−47.5 (3)
N7—Rh2—N5—C11	18.7 (4)	N7—C15—C17—N81	−173.4 (6)
Cl4—Rh2—N5—C11	−167.1 (4)	Rh2—N5—C12—C14	12.4 (11)
Cl6—Rh2—N5—C11	−77.9 (4)	Rh2—N6—C8—C10	43.2 (5)
N6—Rh2—N5—C12	96.3 (6)	C16—N7—C10—C8	−95.7 (4)
N7—Rh2—N5—C12	13.1 (6)	C9—N7—C10—C8	−43.9 (4)
Cl4—Rh2—N5—C12	−172.8 (6)	C14—N7—C10—C8	151.7 (4)
Cl6—Rh2—N5—C12	−83.5 (6)	Rh2—N7—C10—C8	43.1 (4)
N5—Rh2—N6—C7	−92.1 (3)	N6—C8—C10—N7	−57.6 (5)
N7—Rh2—N6—C7	−8.9 (3)	N5—C12—C14—N7	−40.2 (10)
Cl4—Rh2—N6—C7	178.1 (3)	C10—N7—C14—C12	−66.4 (6)
Cl5—Rh2—N6—C7	87.9 (3)	C16—N7—C14—C12	175.3 (6)
N5—Rh2—N6—C8	−98.1 (4)	Rh2—N7—C14—C12	47.9 (6)
N7—Rh2—N6—C8	−14.9 (4)	C10—N7—C16—C18	176.1 (3)
Cl4—Rh2—N6—C8	172.1 (4)	C14—N7—C16—C18	−71.1 (4)
Cl5—Rh2—N6—C8	81.9 (4)	Rh2—N7—C16—C18	42.8 (4)
N6—Rh2—N7—C10	−15.86 (17)	N7—C16—C18—N82	−176.3 (8)
N5—Rh2—N7—C10	79.09 (17)	N2—Rh1—N1—C5	132.69 (9)
Cl6—Rh2—N7—C10	165.41 (16)	N3—Rh1—N1—C5	−135.22 (9)
Cl5—Rh2—N7—C10	−100.08 (16)	Cl3—Rh1—N1—C5	45.47 (8)
N6—Rh2—N7—C13	−83.64 (13)	Cl2—Rh1—N1—C5	−46.51 (8)
N5—Rh2—N7—C13	11.31 (13)	N2—Rh1—N1—C3	−107.53 (9)
Cl6—Rh2—N7—C13	97.63 (12)	N3—Rh1—N1—C3	−15.44 (9)
Cl5—Rh2—N7—C13	−167.86 (12)	Cl3—Rh1—N1—C3	165.25 (8)
N6—Rh2—N7—C15	147.09 (17)	Cl2—Rh1—N1—C3	73.27 (8)
N5—Rh2—N7—C15	−117.96 (17)	N2—Rh1—N1—C1	14.44 (9)
Cl6—Rh2—N7—C15	−31.64 (16)	N3—Rh1—N1—C1	106.54 (9)
Cl5—Rh2—N7—C15	62.87 (16)	Cl3—Rh1—N1—C1	−72.77 (8)
N6—Rh2—N7—C16	118.5 (2)	Cl2—Rh1—N1—C1	−164.76 (8)
N5—Rh2—N7—C16	−146.5 (2)	N3—Rh1—N2—C2	−75.84 (10)
Cl6—Rh2—N7—C16	−60.2 (2)	N1—Rh1—N2—C2	8.76 (9)
Cl5—Rh2—N7—C16	34.3 (2)	Cl1—Rh1—N2—C2	−167.08 (9)
N6—Rh2—N7—C9	29.04 (11)	Cl3—Rh1—N2—C2	103.23 (9)
N5—Rh2—N7—C9	123.98 (11)	N2—Rh1—N3—C4	76.85 (10)
Cl6—Rh2—N7—C9	−149.69 (10)	N1—Rh1—N3—C4	−8.24 (9)
Cl5—Rh2—N7—C9	−55.18 (10)	Cl1—Rh1—N3—C4	167.53 (9)
N6—Rh2—N7—C14	−126.39 (14)	Cl2—Rh1—N3—C4	−101.75 (9)
N5—Rh2—N7—C14	−31.44 (14)	C3—N1—C5—C6	66.79 (15)
Cl6—Rh2—N7—C14	54.89 (13)	C1—N1—C5—C6	−58.10 (15)
Cl5—Rh2—N7—C14	149.40 (13)	Rh1—N1—C5—C6	−175.06 (10)
Rh2—N5—C11—C13	−45.3 (4)	C5—N1—C1—C2	−155.76 (12)
Rh2—N6—C7—C9	−15.9 (5)	C3—N1—C1—C2	81.92 (15)
N6—C7—C9—N7	41.9 (5)	Rh1—N1—C1—C2	−35.48 (14)
C13—N7—C9—C7	67.3 (3)	C5—N1—C3—C4	157.88 (12)
C15—N7—C9—C7	−172.5 (3)	C1—N1—C3—C4	−80.38 (15)
Rh2—N7—C9—C7	−45.9 (3)	Rh1—N1—C3—C4	36.77 (13)
C15—N7—C13—C11	94.9 (5)	Rh1—N3—C4—C3	30.36 (14)
C9—N7—C13—C11	−147.9 (4)	N1—C3—C4—N3	−45.09 (16)
Rh2—N7—C13—C11	−38.6 (4)	Rh1—N2—C2—C1	−30.32 (14)
N5—C11—C13—N7	57.2 (4)	N1—C1—C2—N2	44.48 (17)
C13—N7—C15—C17	−174.9 (2)	N1—C5—C6—N4	−179.70 (12)
C9—N7—C15—C17	67.9 (3)		

### Hydrogen-bond geometry (Å, °)

**Table:**

*D*—H···*A*	*D*—H	H···*A*	*D*···*A*	*D*—H···*A*
N3—H3B···Cl7^i^	0.92	2.42	3.2560 (13)	150.
N3—H3A···Cl4^i^	0.92	2.47	3.3279 (12)	155.
N2—H2B···Cl7^i^	0.92	2.34	3.1744 (12)	151.
N5—H5D···Cl8	0.92	2.45	3.3452 (13)	166.
N4—H4A···Cl8^ii^	0.91	2.26	3.1322 (13)	161.
N5—H5C···Cl4^iii^	0.92	2.75	3.5319 (12)	143.
N6—H6D···Cl1^iv^	0.92	2.62	3.4354 (13)	148.
N4—H4C···Cl6^v^	0.91	2.35	3.1729 (13)	150.
N81—H81B···Cl7^vi^	0.91	2.24	3.109 (14)	159.
O1W—H1WA···Cl7^vii^	0.86	2.25	3.0797 (15)	163.
N2—H2A···Cl1^viii^	0.92	2.62	3.4348 (12)	148.
N6—H6C···Cl8	0.92	2.36	3.2199 (13)	156.

Symmetry codes: (i) −*x*, *y*−1/2, −*z*+1/2; (ii) *x*, −*y*+1/2, *z*−1/2; (iii) −*x*+1, −*y*+1, −*z*+1; (iv) −*x*, *y*+1/2, −*z*+1/2; (v) −*x*+1, *y*−1/2, −*z*+1/2; (vi) −*x*+1, −*y*+1, −*z*; (vii) *x*, −*y*+3/2, *z*+1/2; (viii) −*x*, −*y*, −*z*+1.
